# Module Anchored Network Inference: A Sequential Module-Based Approach to Novel Gene Network Construction from Genomic Expression Data on Human Disease Mechanism

**DOI:** 10.1155/2017/8514071

**Published:** 2017-01-18

**Authors:** Annamalai Muthiah, Susanna R. Keller, Jae K. Lee

**Affiliations:** ^1^Department of Systems and Information Engineering, University of Virginia, Charlottesville, VA 22904, USA; ^2^Department of Medicine, Division of Endocrinology and Metabolism, University of Virginia, Charlottesville, VA 22908, USA; ^3^Department of Biostatistics and Bioinformatics, Moffitt Cancer Center, 12902 Magnolia Drive, Tampa, FL 33612, USA; ^4^Department of Public Health Sciences, University of Virginia School of Medicine, Charlottesville, VA 22908, USA

## Abstract

Different computational approaches have been examined and compared for inferring network relationships from time-series genomic data on human disease mechanisms under the recent Dialogue on Reverse Engineering Assessment and Methods (DREAM) challenge. Many of these approaches infer all possible relationships among all candidate genes, often resulting in extremely crowded candidate network relationships with many more False Positives than True Positives. To overcome this limitation, we introduce a novel approach, Module Anchored Network Inference (MANI), that constructs networks by analyzing sequentially small adjacent building blocks (modules). Using MANI, we inferred a 7-gene adipogenesis network based on time-series gene expression data during adipocyte differentiation. MANI was also applied to infer two 10-gene networks based on time-course perturbation datasets from DREAM3 and DREAM4 challenges. MANI well inferred and distinguished serial, parallel, and time-dependent gene interactions and network cascades in these applications showing a superior performance to other in silico network inference techniques for discovering and reconstructing gene network relationships.

## 1. Introduction

Many established algorithms and approaches are available for inferring gene regulatory networks from large time-course molecular data [1, 2]. In silico network inference challenges under the Dialogue on Reverse Engineering Assessment and Methods (DREAM) projects—DREAM3, DREAM4, and DREAM5—have explored the strengths and weaknesses of important and widely used network inference techniques based on gene expression data. Until recently, in collaboration with the Gene Pattern team at the Broad Institute, the DREAM challenge team had selected successful network inference approaches and made them available as user friendly software algorithms and pipelines of applications that allowed users to combine multiple network inference methods on a platform so-called Gene Pattern-Dialogue on Reverse Engineering Assessment and Methods (GP-DREAM) [2, 3]. Some of the widely used network inference approaches are ANOVerence (which detects gene relationships using nonlinear correlation coefficient derived from an analysis of variance (ANOVA) [4]), correlation (which is based on pairwise correlation between genes [2]), CLR (Context Likelihood of Relatedness, which estimates gene relationships using the concept of mutual information between genes [5]), GENIE3 (which predicts expression profile of each novel gene from expression profiles of Transcription Factors using a tree based ensemble method [6]), Inferelator (network inference approach combining two key time-series data techniques for network inference: time-lagged CLR (tlCLR), an extension of CLR described above, and linear ODE model constrained by LASSO [7]), and TIGRESS (Trustful Inference of Gene Regulation using Stability Selection, a LASSO-based regression approach for inferring gene regulations [8]). Most of these network inference approaches adopt a “global” approach to network inference and construct a network using all genes simultaneously. While it is useful for initial gene network inference, such an approach often produces a* hairball-like* network that makes it hard to discern trustworthy network features among candidate network connections. Similar to the dynamic algorithm in sequence alignment, a localized approach anchoring network inference around building blocks (modules) [1] or subunits of a large network can dramatically enhance computational network reconstruction. Based on this principle, we developed our Module Anchored Network Inference (MANI) technique, which identifies gene interactions and regulatory relationships within each local module and then gradually expands the network by adding new network interactions from adjacent connected modules. This systematic and local approach to network inference constructs a less complex network and identifies dynamic relationships between network genes.

We applied MANI to time-course gene expression data of a 7-gene network during adipocyte differentiation (adipogenesis) [9]. We also tested MANI's ability to infer two small size (*n* = 10) in silico networks based on perturbation time-series data from the DREAM3 and DREAM4 challenges and compared the performance of MANI against contemporary network inference methods such as ANOVerence, CLR, and TIGRESS.

## 2. Methods

### 2.1. MANI Approach

**Figure 1:**
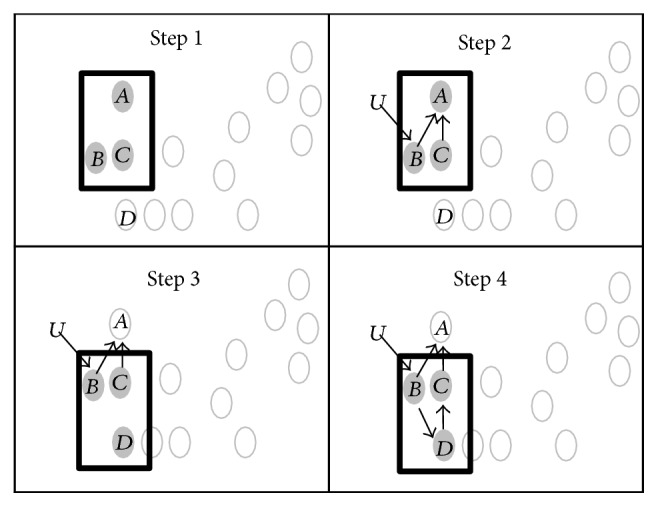
Schematic of MANI steps.  Step 1: selection of three genes, *A*, *B*, and *C*, for the initial window.  Step 2: inferring regulatory relationship among genes within the window as indicated by arrows. External input is shown by *U*.  Step 3: migrating window to accommodate a new gene (*D*) with the closest expression association with two genes (genes *B* and *C*) and their associated regulatory relationships from the previous window.  Step 4: inferring regulatory relationships between the genes in the new window. Steps 3 and 4 are repeated until all the genes in the network are included in a window at least once.

**Figure 2:**
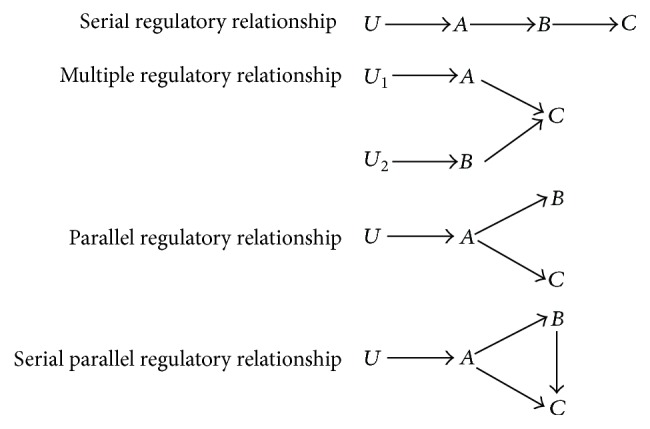
Possible gene regulatory relationships within a three-gene module. The three genes within a MANI module are labeled *A*, *B*, and *C*. The arrows between genes indicate the directions of regulation tested between the genes. External inputs regulating expression of genes are given as *U*, *U*_1_, or *U*_2_.

The goal of MANI is to locally infer gene regulatory relationships with sequential blocks (modules), each containing three genes (shown as a metaphorical window in Figure 1). Our three-gene module network reconstruction approach is based on (i) the observation that majority of regulatory network relationships can be captured by one of the four structures in Figure 2, each of which can be gradually reconstructed with sequential three-gene modules, and (ii) computational network inference can be efficiently performed for all possible relationships within a three-gene module. The approach consists of four steps (Figure 1): (i) identifying a set of three closest genes for the initial window(s), (ii) fitting the best possible mechanism of regulation among the genes within the initial window(s), (iii) migrating the window to next module including a new network gene which has the closest and statistically significant association with the three genes in the previous module and whose relationship has not yet been reconstructed (removing the least associated gene with the new gene among the previous three), and (iv) fitting regulatory relationships between the new gene and the genes retained from the previous module. The last two steps are repeated until all the genes potentially in the network are examined and their relationships are reconstructed if they are determined to be valid network connections based on preset criteria.

### 2.2. Inference of Regulatory Relationship within a Module

A local network module that contains the three most strongly correlated genes was identified by evaluating spearman rank correlations from time-series gene expression profiles. Regulatory relationships between genes within the module are inferred by selecting the optimal gene relationships from a list of possible regulatory relationships (Figure 2).

**Figure 3:**
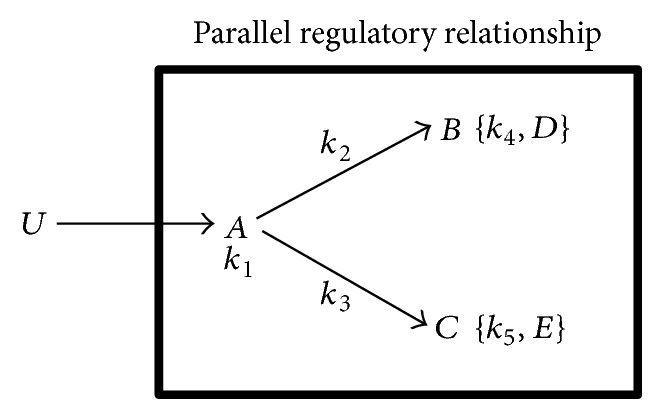
Parameters representing regulatory relationships between three genes (*A*, *B*, and *C*) are given in (1), (2), and (3). Parameters representing the magnitude of regulation of one gene by another (*k*_2_, *k*_3_) are shown over the edge connecting the genes. Rate of degradation of mRNA produced by genes is given within { } ({*k*_4_, *D*} and {*k*_5_, *E*}) or below the names of genes (*k*_1_). *U* represents an external input regulating expression of genes within the module.

Regulatory gene relationships are mathematically modeled and fitted to gene expression data and the optimal relationship is identified using the goodness of fit measure.  Figure 3 and (1)–(3) give an example of a mathematical model for parallel regulatory relationship between genes.

(1) dAdt=U−k1·A,

(2) dBdt=k2·A−k4·B−D,

(3) dCdt=k3·A−k5·C−E.

State variables *A*, *B*, and *C* represent the relative number of mRNA molecules of the respective genes. *U* is a time-invariant input or a sigmoid function that generates a time-delay in the expression of target genes. Parameters of the mathematical model ((1)–(3)) are estimated using the SIMBIOLOGY toolbox within MATLAB R2012b, using the SBIONLINFIT procedure in SIMBIOLOGY by fitting the model to time-series gene expression data and minimizing the Sum of Square of Errors (SSE) of fit for each gene in the module. Similarly, other regulatory relationships shown in Figure 2 are tested on gene expression data. Bayesian Information Criterion (BIC) score estimated for each relationship (by using the aggregate Sum of Square of Errors (SSE) of all three genes as shown below in (6)) is used as the goodness of fit measure to select the optimal regulatory relationship between genes in a module. While testing different regulatory relationships between genes within a module, differences in lags between genes are used to infer hierarchy in gene regulatory relationships assuming that a module gene with a longer lag could be regulated by a gene with a shorter lag (Figure 2). Lags are determined based on a gene's time-series expression profiles to start at *t* = 0 and end at the time point after which gene expression increases or decreases for at least two time points.

### 2.3. Estimation of BIC Score

We use the Bayesian Information Criterion (BIC) (4) as the appropriate goodness of fit measure for selecting optimal regulatory relationships within a module because it accounts for both the number of parameters used in the mathematical model (*p*) to describe the regulatory relationship of genes in a module and the number of time points in the gene expression data (*n*) that is used to estimate the parameters of the model [10].

(4) $$\begin{array}{c} BIC=-2\cdot \ln\left(\;L\;\right)+p\cdot \ln\left(\;n\;\right) \end{array}$$

*L* is the maximized likelihood function of the model describing the regulatory relationship of genes. Summing up the BIC values of all genes in the module (genes *A*, *B*, and *C* in Figure 2), the full BIC score of any regulatory relationship in Figure 2 is

(5) BIC=SSEAτA+SSEBτB+SSECτC+pA·ln⁡nA+pB·ln⁡nB+pC·ln⁡nC.

SSE_*A*_, SSE_*B*_, and SSE_*C*_ represent the SSE of the fitted model for each gene by the regulatory relationship. *τ*_*A*_, *τ*_*B*_, and *τ*_*C*_ represent the standard deviation of error distribution in the fitted model of each gene. The derivation of Likelihood (*L*) in terms of SSE for each gene (SSE_*A*_, SSE_*B*_, and SSE_*C*_) is shown in Supplementary Material (available online at https://doi.org/10.1155/2017/8514071). Since the number of time points in expression data is the same for each gene, *n*_*A*_ = *n*_*B*_ = *n*_*C*_ = *n*. Similarly, since the variance of error for each gene is also approximated to be the same, *τ*_*A*_ = *τ*_*B*_ = *τ*_*C*_ = *τ*. Therefore, the BIC score for a regulatory relationship is

(6) $$\begin{array}{c} BIC=\frac{\left(\sum_{i=1}^{g}{SSE}_{i}\right)}{\tau }+p_{total}\cdot \ln\left(\;n\;\right). \end{array}$$

∑_*i*=1_^*g*^SSE_*i*_ was Sum of Square of Error (SSE) of fit for all the genes within a module (*g* refers to the number of genes within the module) and *p*_total_ = *p*_*A*_ + *p*_*B*_ + *p*_*C*_ represents the total number of parameters in the mathematical model describing the regulatory relationship between genes.

## 3. Results

### 3.1. Implementation of MANI towards Network Inference of a 7-Gene Adipogenesis Network

**Figure 4:**
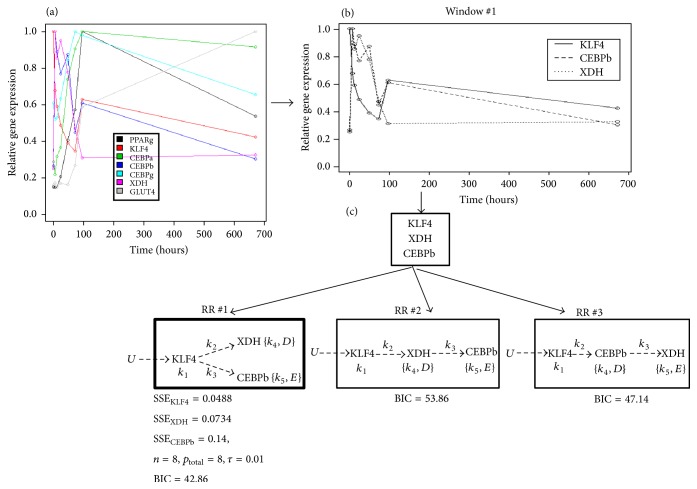
Window #1 network inference. (a) Time-series gene expression data of 7 genes within the adipogenesis network collected at 0, 6, 12, 24, 48, 72, 96, and 672 hours during adipocyte differentiation [9]. GEO accession number of gene expression data is GSE6795. Gene expression values were normalized to a maximum value of 1. Expanded view of gene expression data between 0 and 100 hours is shown in Supplementary Figure S1. (b) Time-series expression profiles of the three genes selected from the pool of 7 genes for window #1. (c) The parameters of different Regulatory Relationships (RRs) were fitted to the time-series data of genes shown in (b). Since KLF4 was determined to be the gene at the top of the hierarchy, the two remaining genes were fitted in parallel or serial regulatory relationships. Parameters describing the different regulatory relationships are as described in Figure 3. BIC scores of regulatory relationships were estimated as (SSE_KLF4_ + SSE_XDH_ + SSE_CEBPb_)/*τ* + *p*_total_ · ln⁡(*n*). Parallel regulatory relationship (RR #1) was chosen as optimal to describe gene relationships in window #1 because of its smallest BIC score. The parameters estimated for the selected regulatory relationship are listed in Table 1.

The MANI approach was implemented on time-series gene expression data obtained from a network of seven genes that belong to an adipogenesis regulatory network [11]: Kruppel Like Factor 4 (KLF4), CCAAT/Enhancer Binding Protein-alpha (CEBPa), CCAAT/Enhancer Binding Protein-beta (CEBPb), CCAAT/Enhancer Binding Protein-gamma (CEBPg), GLUcose Transporter type 4 (GLUT4), Xanthine Dehydrogenase (XDH), and Peroxisome Proliferator-Activated Receptor-gamma (PPARg) (Figure 4(a)). Gene expression data had been collected during differentiation of 3T3-L1 preadipocytes into mature adipocytes for a period of 28 days [9].

**Step 1 (selecting initial window(s)).**

The first two genes in initial windows were selected as the pair(s) of genes with maximum correlation between time-series expression data. A third gene was added by choosing a gene with maximum correlation with either of the genes forming the pair. Among the seven genes (Figure 4(a)), two pairs of genes (pair #1 = (XDH, CEBPb) and pair #2 = (CEBPa, CEBPg)) with the highest degrees of correlation, *ρ* = 0.88, were selected (correlation matrix between genes is shown in Supplementary Table S1). Following our criteria outlined above, a third gene was added to each of the two pairs resulting in window #1 = (KLF4, XDH, and CEBPb) and window #2 = (CEBPa, CEBPg, and PPARg). Expression data of genes selected for window #1 is shown in Figure 4(b).

**Step 2 (fitting the best regulatory relationship for genes within the initial window(s)).**

**Figure 5:**
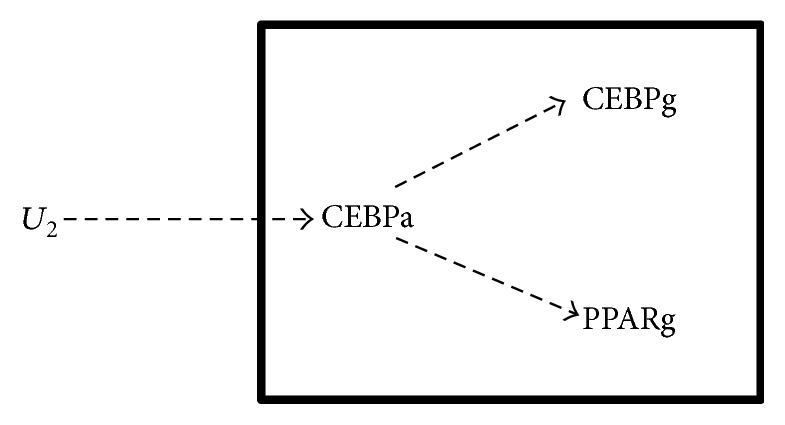
Window #2 optimal regulatory relationship. Expression profiles of all three genes showed nonzero lags (CEBPa (6 hours), CEBPg (6 hours), and PPARg (12 hours)). Between the two genes with shortest lags, CEBPa showed a better fit with external input *U*. The input *U* was a sigmoid function to produce a delayed response in the activity of gene CEBPa. Parallel and serial regulatory relationships were tested and the above optimal regulatory relationship yielded the smallest BIC score.

The possible regulatory relationships between the three genes within a window (listed in Figure 2) were tested. Prior to this, a preliminary check was conducted to determine whether the time-course expression data of genes within a window showed differences in lags. Figure 4(b) shows there were no significant differences in lags between the three genes of window #1. In the absence of lag differences, the top gene in the hierarchy was chosen by testing all three genes (KLF, XDH, and CEBPb) within the window in that position (gene *A*) using (1) with a single time-invariant input (*U*). The various choices of *A* lead to the following results: SSE of fit for the different genes were SSE_KLF4_ = 0.0488, SSE_XDH_ = 0.5220, and SSE_CEBPb_ = 0.3906. Thus, KLF4 was the best fit at the top of the regulatory relationship in window #1. Parallel and serial regulatory relationships were then tested for the other two genes in window #1 as shown in Figure 4(c). Based on estimated BIC scores, the optimal regulatory relationship for genes in window #1 was the parallel regulatory relationship (RR#1). Solving the gene regulatory network for genes in window #2 using the same approach used for window #1, we obtained the inferred network shown in Figure 5.

**Step 3 (migrating the window(s) to accommodate new genes).**

A new gene was introduced into the initial window using a One Gene In, One Gene Out (OIOO) rule. A new gene among the remaining genes outside the window with the highest correlation with any gene inside the current window was identified while the gene least correlated with the new gene was discarded. By keeping at least one gene and its associated interactions from the previous window, we limited the number of possible regulatory relationships with the new gene(s). If introducing a new gene into the window formed an earlier window, the rule was relaxed to include the gene with the next highest degree of correlation with the genes in the window. Window #1 was thus advanced by replacing gene XDH with gene CEBPg as correlation of CEBPg with KLF4 (*ρ* = 0.72, Supplementary Table S1) was highest and XDH was least correlated with CEBPg (*ρ* = 0.03). New window #3 thus contained KLF4, CEBPb, and CEBPg. Similarly window #2 (CEBPa, CEBPg, and PPARg) was migrated to window #4 (GLUT4, PPARg, and CEBPa) by replacing CEBPg with GLUT4.

**Step 4 (fitting the regulatory relationships within the new window(s)).**

**Figure 6:**
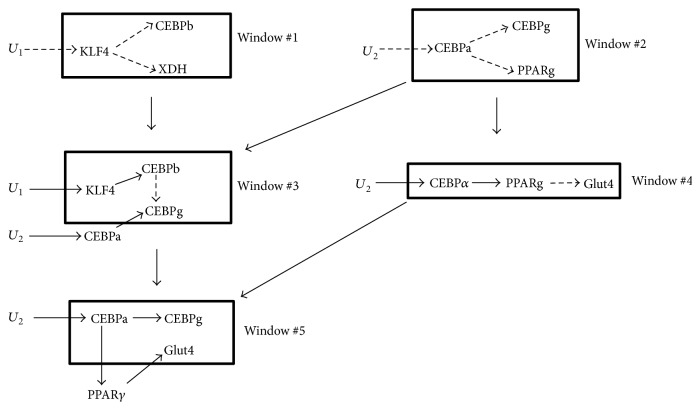
Windows of 7-gene adipogenesis network. All windows covering the 7-gene network are shown. Newly inferred gene interactions inside the window are indicated by broken arrows while interactions inferred from a previous window are indicated by solid arrows. Window #5 did not have any broken arrows connecting genes because no new gene relationships were inferred; the null hypothesis was the optimal regulatory relationship connecting genes. Furthermore, windows contributing gene relationships to other windows are shown by solid arrows between windows.

For the new genes in the newly created windows, regulatory relationships were inferred while retaining genes and their associations from previous windows. For example, in window #3, the regulatory relation of the new gene in the window, CEBPg, was tested taking into account gene relationships to KLF4 and CEBPb from window #1. The time-course expression profiles of genes in window #3 indicated a noticeable lag for CEBPg when compared to genes KL4 and CEBPb (Figure 4(a), Supplementary Figure S1). Thus, regulatory relationships tested potential regulation of CEBPg by KLF4 and/or CEBPb. Since CEBPg was already inferred to be regulated by gene CEBPa from window #2 (Figure 5), regulatory relationships tested in window #3 included this regulatory interaction. While fitting potential gene relationships in the new windows, an additional alternate relationship was also tested, the null hypothesis scenario. The null hypothesis scenario introduces no new regulatory edges between genes in the window to prevent overfitting. The new inferred regulatory relationships for windows #3 and #4 are shown in Figure 6. MANI Steps 3 and 4 were repeated until all the 7 genes in the adipogenesis network were covered at least once by the moving windows. In total, 5 windows were created and gene relationships inferred within each window are shown in Figure 6.

**Figure 7:**
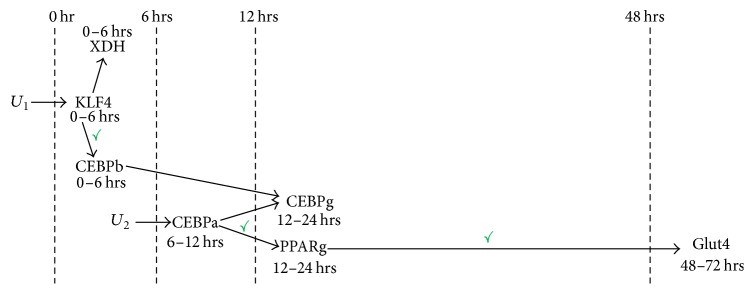
Dynamic adipogenesis network constructed by MANI. The two inputs of the network were *U*_1_ (time-invariant constant input) and *U*_2_ (sigmoid input). The network, besides representing gene relationships collected from MANI windows in Figure 6, was also organized according to the genes' likely times of activation in the cascade. A gene's time of activation in the cascade was derived from a gene's lag in its time-series expression data. Based on the ranges of times of activation in the cascade, genes were grouped in appropriate time zones in the cascade (marked by dashed vertical lines). Arranging genes in such a manner enhanced the dynamical nature of the network. Gene relationships in this MANI constructed adipogenesis network that were verified using literature are indicated with a green tick mark.

The cumulative adipogenesis network inferred by MANI through the 5 windows is shown in Figure 7. In addition to the gene relationships summarized from the various windows, a gene's likely time of activation, derived from the gene's lag observed in the time-series expression data (Supplementary Figure S1), was included in the network. Some of the inferred gene relationships were supported by the literature. KLF4 regulates the expression of CEBPb [12] and PPARg regulates the expression of GLUT4 [13]. Fibroblasts isolated from C/EBPa−/− embryos have reduced PPARg levels and do not differentiate well when exposed to hormonal inducing agents in culture [14], implying regulation of PPARg by CEBPa. Indeed later research showed that CEBPa and PPARg regulate each other's expression in a positive feedback loop and PPARg and CEBPa act synergistically to activate expression of fat cell specific genes such as GLUT4 [11].

### 3.2. Validation of MANI Approach

**Figure 8:**
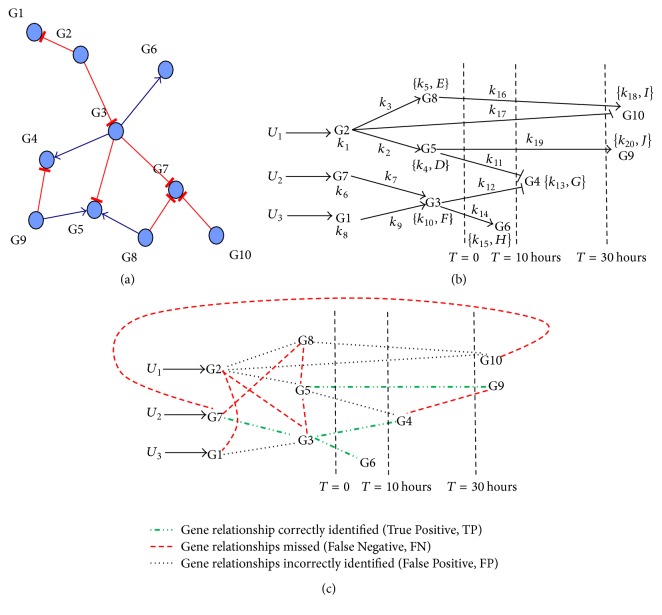
Comparison of MANI inferred DREAM3 network with the correct answer. (a) The 10-gene DREAM3 network that was perturbed by DREAM3 organizers to produce the time- series data. (b) Network inference by MANI. *U*_1_, *U*_2_, and *U*_3_ were external inputs. The edges inferred between genes were either stimulating (→) or inhibitory (⊣). The parameters for the different regulatory relationships followed the same convention described in Figure 3. Genes in the network were grouped according to their likely times of activation within the cascade as estimated from the durations of lag observed in their expression data. (c) The accuracy of the DREAM3 network inferred by the MANI approach in (b) was evaluated by comparing it to the correct answer shown in (a). Comparisons were based on the presence or absence of edges between genes rather than directions of interactions. Among the MANI inferred edges in (b), there were 4 TPs, 7 FNs, 6 FPs, and 28 True Negatives (TNs).

An objective validation of MANI's performance in network inference was conducted using time-series expression data made available as part of the DREAM3 challenge (Supplementary Figure S2). This data was generated by the challenge organizers by perturbing an in silico network of 10 genes derived from* E. coli* (Figure 8(a)). The correlation matrix generated between genes using the time-series data is shown in Supplementary Table S2. The network inferred after applying MANI's gradual and module-based local network inference approach on the DREAM3 time-series perturbation data is shown in Figure 8(b). For bigger networks of genes (number of genes in the network (*N*) ≥ 10 genes), gradual network inference by MANI leads to selection of several windows of genes. In the interest of constructing a parsimonious network, the number of windows is reduced by organizing the selected windows in decreasing order for average degrees of correlation between genes within the window and choosing only those windows from the top where a new gene in the network is selected for the first time in a window. The network is then constructed by inferring relationships of genes through the chosen windows. Based on this principle, the DREAM3 network was inferred by MANI using 8 windows of genes chosen from an initial list of 37 windows. The complete list of MANI selected gene windows and those windows that were chosen to infer DREAM3 network are shown in Supplementary Table S3. The values of the kinetic parameters estimated for the inferred network are given in Supplementary Table S4. The inferred network was compared to the correct DREAM3 network and the accuracy of network inference by MANI was evaluated by classifying MANI inferred edges as True Positives (TPs), False Positives (FPs), True Negatives (TNs), and False Negatives (FNs) (Figure 8(c)). The performance of MANI in network inference was compared against the performance of three other contemporary network inference approaches (ANOVerence, CLR, and TIGRESS) using the same time-series data.

Since our goal was to infer a sparse network and MANI inferred 10 edges between genes, the top 10 edges inferred by each of the methods were used for comparison.  Table 2 shows the performance of our MANI approach in network inference when compared to ANOVerence and CLR. The results for TIGRESS were similar to ANOVerence and CLR. MANI outperforms the other methods in all performance criteria. PPV of MANI with 40% is better than random network prediction as the chance of obtaining 4 correct edges and 6 wrong edges by random guesswork ((^11^*c*_4_ × ^79^*c*_6_)/(^90^*c*_10_)) is low (0.02). The same principles were applied to construct a size 10 network (Supplementary Table S5) from the DREAM4 challenge using two sets of perturbation data (Supplementary Figure S3). The performance of MANI (sensitivity ~27%) was comparable to TIGRESS (~33%) but worse than that of CLR (~47%).

## 4. Discussion

Gene expression data are generated in biological experiments at an increasing rate for the purpose of studying complex gene regulatory mechanisms and human disease mechanisms [15–17]. Collection of time-series gene expression data has become important to deduce causal regulatory relationships between genes belonging to a network [18]. A number of network inference methods to infer gene regulatory networks from time-series gene expression data have been developed. These include solving linear ODE regression models, inferring optimal regulatory relationships between genes through a combination of procedures such as variable selection and sparse network identification, using shrinkage techniques such as LASSO and SCAD [19], solving ODE regression models to obtain possible solutions by Singular Value Decomposition followed by selection of a parsimonious network, and using various multivariate modeling techniques such as robust regression [20, 21], Dynamic Bayesian Network modeling [22–24], and time-delayed ARCANE algorithm that infers gene relationships based on mutual information between genes [25]. The common feature of the currently used methods is that they generate a global network of gene relationships in an unsupervised manner. Therefore, the constructed network is often crowded and does not provide a clear delineation of the network's hierarchical or dynamical features (e.g., Figure 8(a)). With increasing numbers of genes in the network, the complexity of interconnections between genes increases exponentially, making in silico reconstruction of the network rapidly intractable.

In contrast, MANI adopts a systematic and gradual approach to network inference by constructing networks within local modules. This local approach to network inference adopted by MANI allowed the final constructed adipogenesis network (Figure 7) to be sparse and well organized, highlighting structural aspects of the network such as the hierarchy in gene relationships and also providing clarity to the network's pathways of activation. MANI's inference of the adipogenesis network in Figure 7 shows that the network follows a serial-parallel pathway for cascade activation. MANI was successful in inferring a hierarchy of regulation between genes when a difference in lag was detected in gene expression profiles. Regulatory relationships between genes G3, G4, G5, G6, and G9 in the DREAM3 network (Figure 8(c)) and genes CEBPa, PPARg, and GLUT4 in the adipogenesis network (Figure 7) were successfully inferred. Adding times of activation of genes to the MANI constructed network enhanced the overall quality of the inferred network by making it more dynamic and interpretable. For example, in the case of the adipogenesis network in Figure 7, arranging genes according to their times of activation showed how the genes in the network switched on at various time intervals. Therefore, MANI inference provided a structural organization of genes in the network. The accuracy of the local approach towards constructing networks proposed by MANI was modestly better than that of other well-known global network inference methods in the DREAM3 challenge (Table 2). The reason for MANI's lower performance in the DREAM4 challenge was due to the presence of feedback loops in the network while MANI has been primarily developed to infer networks without such feedback structures. We believe MANI's approach still has high value in identifying novel biological networks without such loop connections. MANI distinguishes itself from other global network inference approaches in that it can locally yet dynamically reconstruct networks across moving modules and windows and can easily be extended to reconstruct much larger networks. While constructing larger networks using MANI, the inferred network is a local optimum (instead of global) since MANI infers the network using locally constructed network modules, and then additional edges in the network are gradually expanded from neighboring modules. In this regard, MANI is also one of the heuristic algorithms, following a search path based on high probability regulatory expression association of network genes.

We note that the current MANI approach also has several limitations. Inference of hierarchy in the network relies on differences in lags between the expressions of different genes. Therefore, lack of differences in lags between genes hinders MANI's ability to infer regulatory relationships between genes. Relationships between genes G1, G2, G5, and G8 in the DREAM3 network (Figure 8(c)) were incorrectly inferred due to the lack of differences in lags between them in the time-series expression data that was used to construct the network. MANI also currently relies on a constant or sigmoid perturbation rate (*U*) in our ODE model for network inference, which can be relaxed in a future study. Time-series data obtained by a single perturbation of the network may also activate multiple genes within the network and, therefore, in order to maximize network inference performance by MANI, multiple time-series data generated by multiple perturbations of the same network can be used for improved network inference to distinguish such multiple interactions. Furthermore, the current ODE model of MANI is best suited for time-series gene expression data to infer gene regulatory networks. MANI's scope can be expanded by developing a local approach to network inference using static gene expression data in future applications.

## Supplementary Material

Supplementary figures provide the time series gene expression data to which MANI and other gene network inference techniques were applied with adipogenesis gene expression data shown in Supplemental Figure S1, and DREAM3 and DREAM4 gene expression data in Supplemental Figures S2 and S3, respectively. The supplementary tables provide the correlation matrices between genes in the adipogenesis network (Supplementary Table S1) and DREAM3 network (Supplementary Table S2). These matrices helped create the modules (metaphorical gene windows) based on which the networks were constructed. The gene windows and the kinetic parameters estimated by MANI for the DREAM3 network are shown in Supplementary Tables S3 and S4, respectively. The true regulatory relationships between genes in the DREAM4 network are listed in Supplementary Table S5. Furthermore, the derivation of the formula to estimate the likelihood that a certain mathematical model captures the gene regulatory relationship between three genes in a given window is described in the supplementary material.

## Figures and Tables

**Table 1:** Values of kinetic parameters for regulatory relationship in window #1 (RR #1 in Figure 4(c)) obtained by fitting mathematical model ((1), (2), and (3)) to gene expression data in Figure 4(b).

Parameters	Mean ± standard error (hr^−1^)
*D*	0.12 ± 0.02
*E*	0.1 ± 0.02
*k*_1_	0.13 ± 0.05
*k*_2_	0.28 ± 0.04
*k*_3_	0.24 ± 0.05
*k*_4_	0.03 ± 0.01
*k*_5_	0.02 ± 0.01
*U*	0.06 ± 0.03

**Table 2:** Network inference performance of MANI and other methods.

Parameters of assessment	ANOVerence	CLR	MANI
Sensitivity^a^	27.27%	27.27%	36.36%
Specificity^b^	79.41%	79.41%	82.35%
PPV^c^	30%	30%	40%

^a^Sensitivity was TP/(TP + FN). ^b^Specificity was TN/(TN + FP). ^c^Positive Predictive Value (PPV) was TP/(TP + FP).
