# Supplementary material for: Module Anchored Network Inference: A Sequential Module-Based Approach to Novel Gene Network Construction from Genomic Expression Data on Human Disease Mechanism
Source: Int J Genomics. 2017 Jan 18;2017:8514071. doi: 10.1155/2017/8514071 (PMC5286469; doi:10.1155/2017/8514071)
Supplement: Supplementary file 1 — Supplementary figures provide the time series gene expression data to which MANI and other gene network inference techniques were applied with adipogenesis gene expression data shown in Supplemental Figure S1, and DREAM3 and DREAM4 gene expression data in Supplemental Figures S2 and S3, respectively. The supplementary tables provide the correlation matrices between genes in the adipogenesis network (Supplementary Table S1) and DREAM3 network (Supplementary Table S2). These matrices helped create the modules (metaphorical gene windows) based on which the networks were constructed. The gene windows and the kinetic parameters estimated by MANI for the DREAM3 network are shown in Supplementary Tables S3 and S4, respectively. The true regulatory relationships between genes in the DREAM4 network are listed in Supplementary Table S5. Furthermore, the derivation of the formula to estimate the likelihood that a certain mathematical model captures the gene regulatory relationship between three genes in a given window is described in the supplementary material. [file 8514071.f1.docx]

**Supplementary Material**


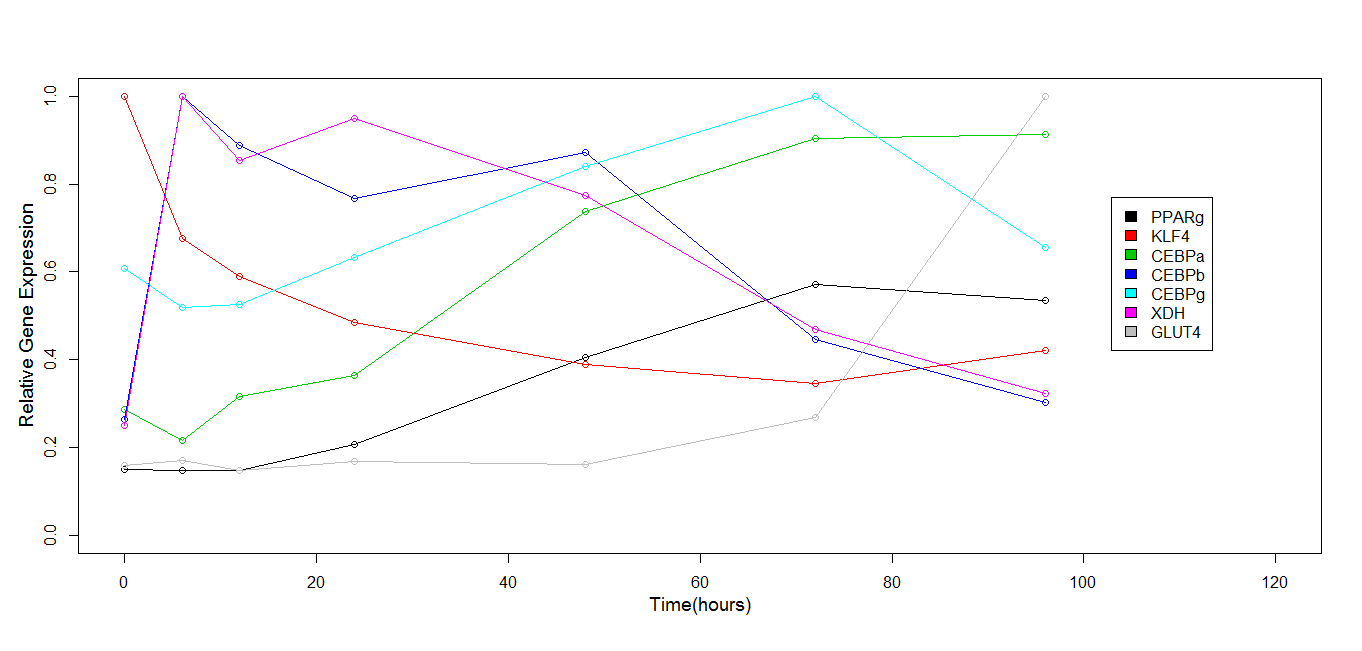


**Supplementary Fig. S1.** Gene expression data in **Figure 4a** magnified between 0 and 100 hours.


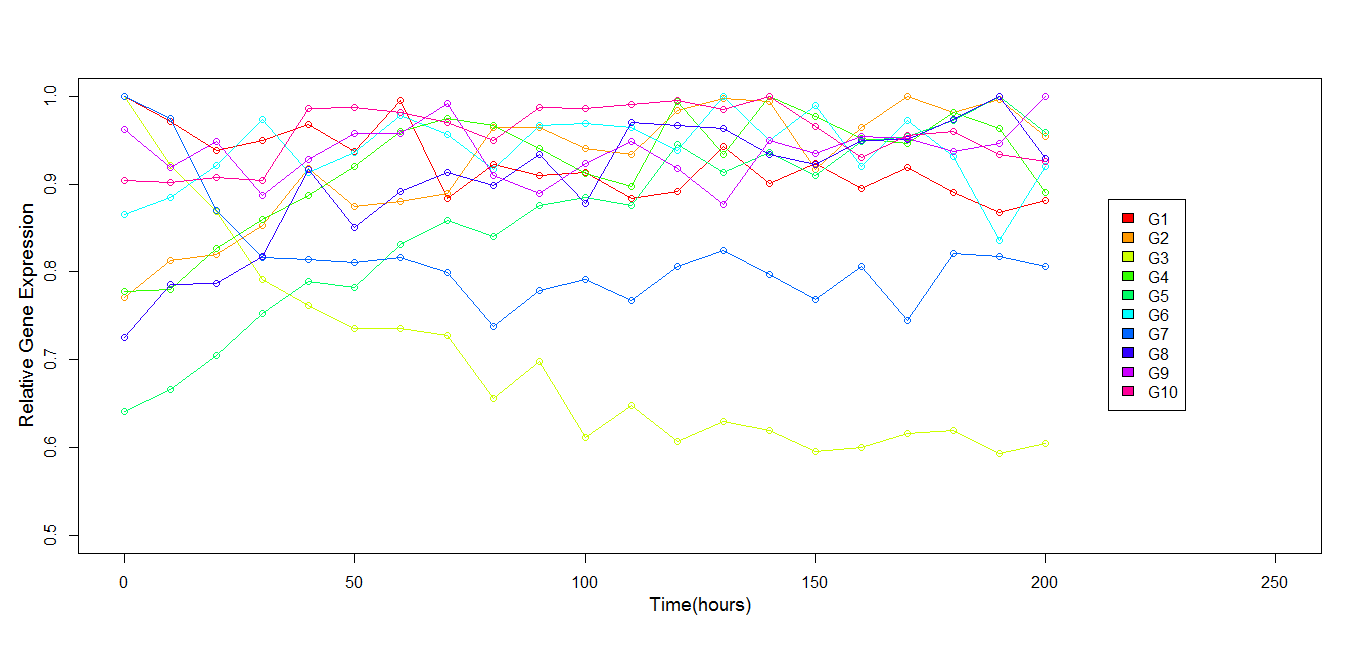


**Supplementary Fig. S2.** DREAM3 Time Series gene expression data. Data was obtained by perturbing a 10-gene network (**Figure 8a**) and collecting the activity of genes for 21 time points in 10 hour intervals. The data was normalized such that maximum possible gene activity was 1.


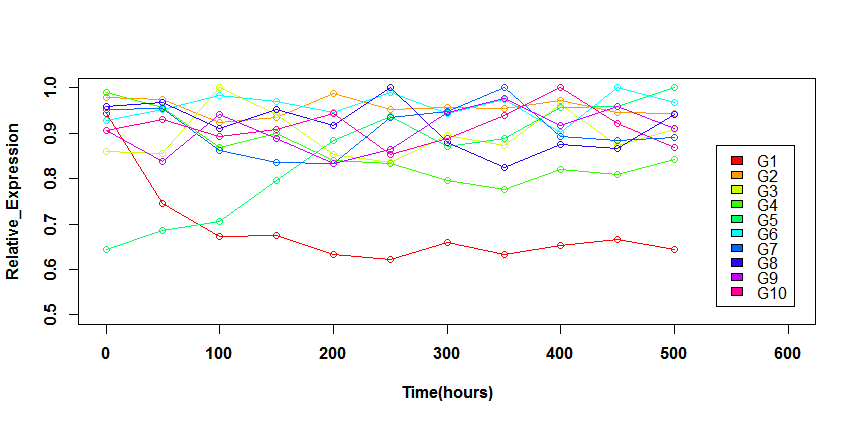


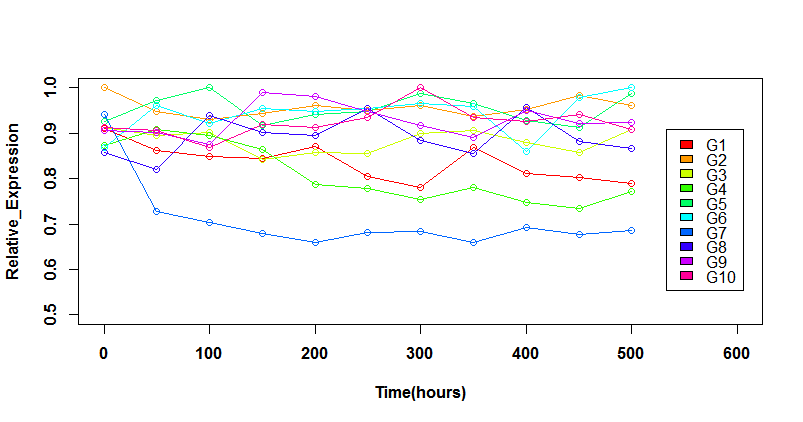


**Supplementary Fig. S3.** DREAM4 Time Series gene expression data. The top and bottom panels represent data obtained by perturbing a 10-gene network (**Sup Table S5**) by two separate perturbations and collecting the activity of genes for the next 10 time points in 50 hour intervals. The data was normalized such that maximum possible gene activity was 1.

**Supplementary Table S1.** Correlation matrix of 7 genes in the adipogenesis network estimated from Time Series expression data (**Figure 4a**)

|  | **PPARg** | **KLF4** | **CEBPa** | **CEBPb** | **CEBPg** | **XDH** | **GLUT4** |
| --- | --- | --- | --- | --- | --- | --- | --- |
| **PPARg** |  | 0.53 | 0.87 | 0.30 | 0.85 | 0.32 | 0.75 |
| **KLF4** |  |  | 0.63 | 0.27 | 0.72 | 0.37 | 0.42 |
| **CEBPa** |  |  |  | 0.13 | 0.88 | 0.17 | 0.67 |
| **CEBPb** |  |  |  |  | 0.07 | 0.88 | 0.05 |
| **CEBPg** |  |  |  |  |  | 0.03 | 0.63 |
| **XDH** |  |  |  |  |  |  | 0.05 |
| **GLUT4** |  |  |  |  |  |  |  |

**Supplementary Table S2.** Correlation matrix generated for the 10 genes within the DREAM3 network based on Time Series expression data shown in **Supplementary Figure S2.**

|  | **G1** | **G2** | **G3** | **G4** | **G5** | **G6** | **G7** | **G8** | **G9** | **G10** |
| --- | --- | --- | --- | --- | --- | --- | --- | --- | --- | --- |
| **G1** |  | 0.56 | 0.71 | 0.50 | 0.76 | 0.1 | 0.43 | 0.72 | 0.19 | 0.27 |
| **G2** |  |  | 0.71 | 0.61 | 0.85 | 0.17 | 0.4 | 0.85 | 0.24 | 0.41 |
| **G3** |  |  |  | 0.62 | 0.91 | 0.15 | 0.45 | 0.74 | 0.02 | 0.24 |
| **G4** |  |  |  |  | 0.63 | 0.25 | 0.44 | 0.60 | 0.01 | 0.49 |
| **G5** |  |  |  |  |  | 0.1 | 0.32 | 0.86 | 0.05 | 0.23 |
| **G6** |  |  |  |  |  |  | 0.36 | 0.12 | 0.24 | 0.42 |
| **G7** |  |  |  |  |  |  |  | 0.3 | 0.06 | 0.49 |
| **G8** |  |  |  |  |  |  |  |  | 0.11 | 0.42 |
| **G9** |  |  |  |  |  |  |  |  |  | 0.12 |
| **G10** |  |  |  |  |  |  |  |  |  |  |

**Supplementary Table S3.** Complete list of gene windows selected by MANI arranged according to their average degrees of correlation between genes within windows. The windows chosen for network inference are highlighted in bold. A, B and C are names of genes within a generic three gene window.

| **Window #** | **A** | **B** | **C** | **Average Correlation** |
| --- | --- | --- | --- | --- |
| **1** | **G5** | **G8** | **G2** | **0.85** |
| **2** | **G3** | **G5** | **G8** | **0.84** |
| 3 | G3 | G5 | G2 | 0.82 |
| **4** | **G3** | **G5** | **G1** | **0.79** |
| 5 | G5 | G8 | G1 | 0.78 |
| 6 | G8 | G2 | G3 | 0.76 |
| 7 | G2 | G5 | G1 | 0.72 |
| 8 | G8 | G3 | G1 | 0.72 |
| **9** | **G3** | **G4** | **G5** | **0.72** |
| 10 | G2 | G1 | G8 | 0.71 |
| 11 | G5 | G8 | G4 | 0.7 |
| 12 | G2 | G4 | G5 | 0.7 |
| 13 | G8 | G4 | G2 | 0.69 |
| 14 | G3 | G1 | G2 | 0.66 |
| 15 | G8 | G3 | G4 | 0.65 |
| 16 | G3 | G2 | G4 | 0.65 |
| **17** | **G7** | **G3** | **G5** | **0.56** |
| **18** | **G2** | **G8** | **G10** | **0.56** |
| 19 | G3 | G1 | G7 | 0.53 |
| 20 | G2 | G7 | G5 | 0.52 |
| 21 | G2 | G7 | G8 | 0.52 |
| 22 | G2 | G4 | G10 | 0.50 |
| 23 | G8 | G4 | G10 | 0.50 |
| 24 | G3 | G4 | G7 | 0.50 |
| 25 | G7 | G8 | G3 | 0.49 |
| 26 | G7 | G8 | G5 | 0.49 |
| 27 | G2 | G1 | G7 | 0.46 |
| 28 | G3 | G10 | G8 | 0.46 |
| 29 | G3 | G10 | G5 | 0.46 |
| 30 | G2 | G10 | G7 | 0.43 |
| 31 | G7 | G8 | G10 | 0.40 |
| 32 | G7 | G3 | G10 | 0.39 |
| **33** | **G7** | **G3** | **G6** | **0.32** |
| 34 | G2 | G7 | G6 | 0.31 |
| 35 | G3 | G10 | G6 | 0.27 |
| 36 | G7 | G8 | G6 | 0.26 |
| **37** | **G10** | **G5** | **G9** | **0.13** |

**Supplementary Table S4.** Kinetic parameters of regulation estimated for the MANI inferred DREAM3 network (**Figure 8b**)

| **Parameters** | **Mean +/- Standard Error**  **(hr^-1^)** | **Parameters** | **Mean +/- Standard Error**  **(hr^-1^)** |
| --- | --- | --- | --- |
| U_1_ | 0.12 ± 0.03 | F | 7.64 ± 1.1 |
| k_1_ | 0.12 ± 0.03 | k_11_ | -2.15 ± 0.51 |
| k_2_ | 2.44 ± 0.71 | k_12_ | -3.0 ± 0.02 |
| k_3_ | 0.92 ± 0.67 | k_13_ | 2.27 ± 0.81 |
| k_4_ | 1.43 ± 0.39 | G | -6.1 ± 0.37 |
| k_5_ | 0.93 ± 0.68 | k_14_ | 0.07 ± 0.01 |
| D | 1.00 ± 0.32 | k_15_ | 0.05 ± 0.01 |
| E | 0 | H | 0 |
| U_2_ | 0.10 ± 0.04 | k_16_ | 3.66 ± 1.96 |
| k_6_ | 0.3 ± 0.07 | k_17_ | -4.06 ± 2.08 |
| U_3_ | 0.24 ± 0.06 | k_18_ | 0.42 ± 0.33 |
| k_8_ | 0.11 ± 0.05 | I | -0.83 ± 0.44 |
| k_7_ | 3.78 ± 1.24 | k_19_ | 0.04 ± 0.02 |
| k_9_ | 6.85 ± 2.26 | k_20_ | 0.19 ± 0.39 |
| k_10_ | 2.81 ± 0.11 | J | -0.15 ± 0.36 |

**Supplementary Table S5.** Gene regulatory relationships in the 10 gene DREAM4 network. The gene in the first column has regulatory control over gene in the second column.

| **Gene 1** | **Gene 2** |
| --- | --- |
| G1 | G2 |
| G1 | G3 |
| G1 | G4 |
| G1 | G5 |
| G3 | G4 |
| G3 | G7 |
| G4 | G3 |
| G6 | G2 |
| G7 | G3 |
| G7 | G4 |
| G8 | G2 |
| G8 | G6 |
| G9 | G10 |
| G10 | G3 |
| G10 | G4 |

**Derivation of Likelihood (L) for mathematical models of gene regulation**

For any gene regulatory relationship X in **Figure 2**, estimation of posterior probability in Bayesian theory is proportional to the product of likelihood and prior probability. Applying that principle,

P (X/Gene Expression Data) α P(Gene Expression Data/ X) P(X)

Therefore, Likelihood (L) of obtaining gene expression from regulatory relationship X is given by P(Gene Expression Data/X). Though we illustrate below how to estimate L for a parallel regulatory relationship between genes within a module, it can be applied to other relationships in **Figure 2** as well.

Based on parallel regulatory relationship shown in **Figure 3**, K is a vector of regulatory parameters [U, k_1_, k_2_, k_3_, k_4_, k_5_, D, E] and G is a matrix composed of Time Series gene expression data of genes A, B and C. Therefore,

L= P(Gene Expression Data/ X) = P(G/X)

L = P(G/X)= P(G/K)=P(A,B,C/K)=P(A/U, k_1_). P(B/U, k_1_, k_2,_ k_4_, D). P(C/U, k_1_, k_3,_ k_5_, E)

= P(ε_A_/U, k_1_). P(ε_B_/U, k_1_, k_2,_ k_4_, D). P(ε_c_/U, k_1_, k_3,_ k_5_, E), (1) where ε_A_, ε_B_ and ε_C_ are random variables representing errors in fits of genes A, B and C.

The error variables (ε_A_, ε_B_ and ε_C_) followed a normal distribution, that is, ε_i_ ~ N(0,$\tau$_i_), where $\tau$_i_  represents the standard deviation of the error distribution. Therefore, applying error distribution in the equation for L (equation #1), we get


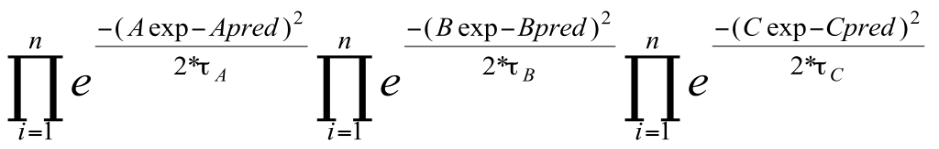


L = (2)

Where A_exp_, B_exp_, C_exp_ are the observed values of expression of genes A, B and C respectively while A_pred_, B_pred_ and C_pred_ are their predicted values by the model representing the regulatory relationship and n is the number of time points in the gene expression data.

Simplifying equation #2, we get

-ln L = (SSE_A_**/** $\tau$_A_+ SSE_B_**/**$\tau$_B_ + SSE_C_**/** $\tau$_C_)/2, where SSE is sum of square of errors of fit (3)
